# Takayasu arteritis: A rare cause of chronic headache

**DOI:** 10.1002/ccr3.4860

**Published:** 2021-09-21

**Authors:** Linda Barasa, Adil Salyani, Jillo Bika, Fredrick Otieno, Dilraj Sokhi

**Affiliations:** ^1^ Department of Medicine Aga Khan University Medical College of East Africa (Nairobi Campus) Nairobi Kenya; ^2^ Department of Radiology Aga Khan University Medical College of East Africa (Nairobi Campus) Nairobi Kenya

**Keywords:** chronic headache, secondary headache, Takayasu arteritis, vasculitis

## Abstract

Chronic headache can be a presenting manifestation of Takayasu arteritis, although patients usually have other characteristic features. A thorough clinical assessment remains key in the evaluation of chronic headache.

## Graphical abstract

Careful history taking and physical examination are invaluable in diagnosis of secondary causes of chronic headache.

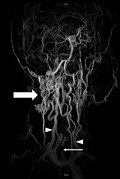

## 1 CASE SUMMARY

A 40‐year‐old African man presented with a 10‐year history of unrelenting, severe (International Headache Society [HIS] category V‐B), bifrontal, throbbing headaches with photophobia and nausea, unresponsive to regular analgesia. He had one prior episode of transient unilateral visual loss, syncope, six‐kilogram weight loss over a year, and left arm claudication. Examination revealed a systolic blood pressure difference of 20 mmHg between arms, a weak left radial pulse, and bruits over left carotid and subclavian arteries. There were no neurological deficits.

**FIGURE 1:**
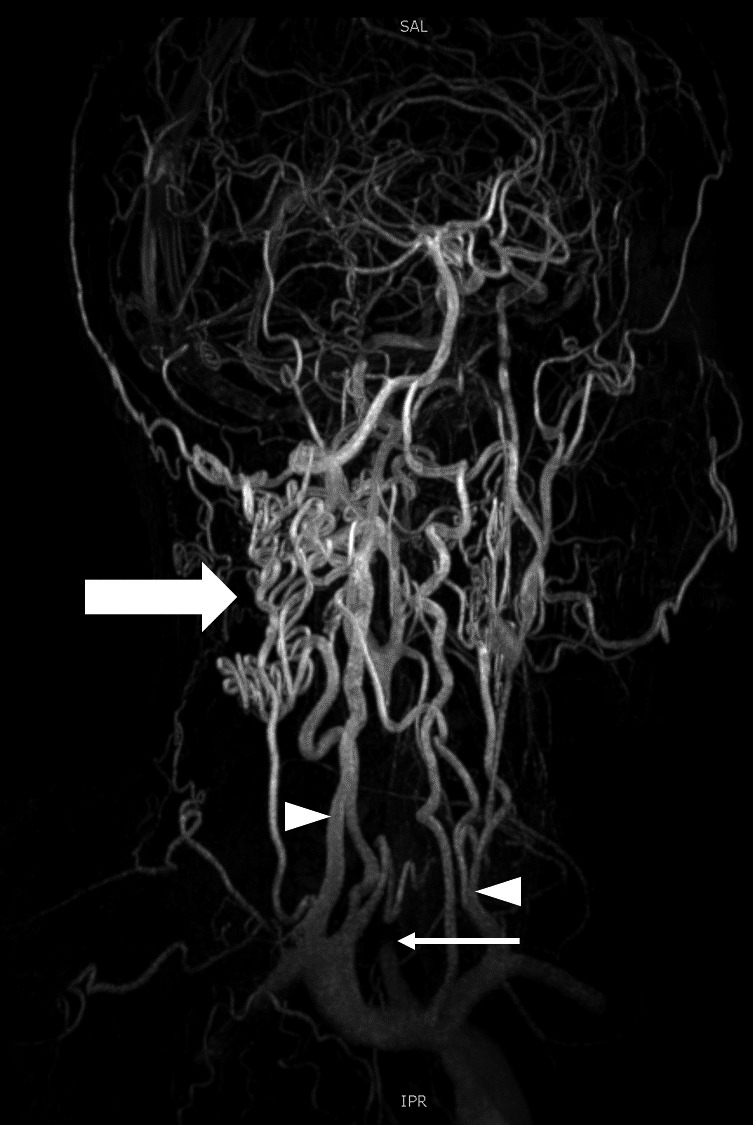
Contrast‐enhanced MRI angiography, maximum intensity projection (MIP) images of the neck arterial system. The vertebral arteries are prominent (white arrowheads) with numerous collaterals in the occipital region and prominence of the posterior circulation (white block arrow). Occlusion of the right common carotid artery is seen (white arrow)

**FIGURE 2:**
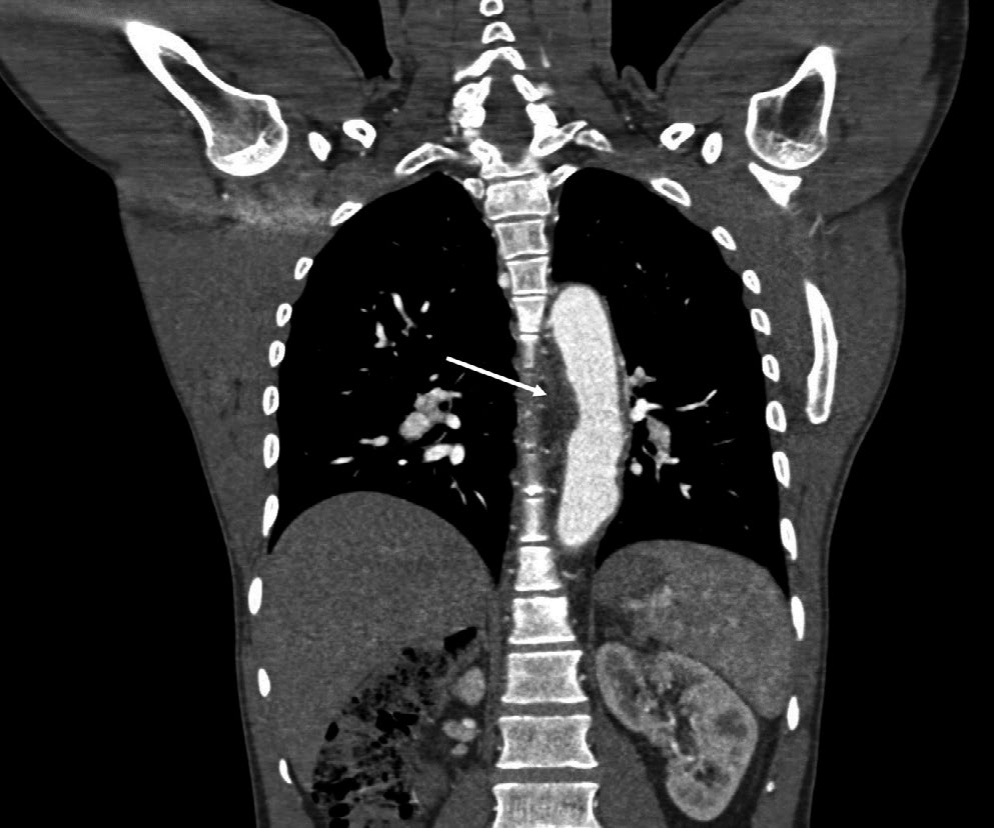
CT aortic angiography coronal reformatted image showing diffuse irregular thickening of the thoracic aorta (white arrow)

Brain magnetic resonance (MR) imaging demonstrated a chronic left insular and inferior frontal infarct. MR angiography revealed complete occlusion of the right common carotid artery and marked stenosis of the left common carotid artery. (Figure 1). CT aortography showed diffuse wall thickening and dilatation of the thoracic aorta (Figure 2). A diagnosis of Takayasu arteritis was made, meeting the American College of Rheumatology (ACR) criteria. He was started on prednisone 40mg once daily and azathioprine 100mg once daily with resolution of the headaches (IHS category I).

Takayasu arteritis may present with neurologic manifestations such as dizziness, visual disturbances, ischemic strokes, and headaches in up to 30% of cases,^1^ possibly due to reduced cerebral blood flow.^1^, ^2^ Vasculitis, while rare, should be kept in mind while evaluating secondary headaches.

## CONFLICTS OF INTEREST

The authors have no conflicts of interest to declare.

## AUTHOR CONTRIBUTIONS

Linda Barasa, Adil Salyani, and Jillo Bika: Conceptualization, Writing—original draft, Writing—Review & Editing, and Visualization. Fredrick Otieno and Dilraj Sokhi: Conceptualization, Writing—review & editing, Visualization, and Supervision.

## ETHICAL APPROVAL

Our work has been conducted in accordance with the Declaration of Helsinki (1964). We have obtained written consent from the patient to publish his case and images. In line with our Institutional Ethics and Research Committee (IERC) guidelines, this case report was exempted from full IERC review.

## AUTHOR STATEMENT

This manuscript is original work and has not been submitted or is not under consideration for publication elsewhere. All the authors have reviewed the manuscript and approved it before submission.

## CONSENT

Obtained from the patient.

## Data Availability

The clinical information and imaging data used to support the findings of this study are included within the article. According to our institutional information governance regulations, the anonymised data can be requested from the corresponding author.
